# AI-assisted point of care ultrasound and an innovative vaginal speculum: a proposed combined approach to reducing spontaneous preterm birth

**DOI:** 10.3389/fmed.2026.1785103

**Published:** 2026-04-24

**Authors:** Jean M. Bouquet, Brooke Emery, Krista R. Fruehauf, Keymia Ghodrati, Emily Fitch, Isain Zapata

**Affiliations:** 1 Department of Primary Care Medicine, Rocky Vista University College of Osteopathic Medicine, Englewood, CO, United States; 2 Rocky Vista University College of Osteopathic Medicine, Englewood, CO, United States; 3 Viospex, Parker, CO, United States; 4 Department of Biomedical Sciences, Rocky Vista University College of Osteopathic Medicine, Englewood, CO, United States; 5 Office of Research and Scholarly Activity, Rocky Vista University, Englewood, CO, United States

**Keywords:** AI-assisted imaging, cervical cerclage, cervical examination, cervical visualization, gynecologic examinations

## Abstract

The Bouquet Speculum^™^ is an innovative vaginal speculum developed to enhance patient comfort, improve cervical visualization, and facilitate ease of use for healthcare providers during gynecologic examinations and procedures. Studies show AI-assisted ultrasound can identify women at risk for spontaneous preterm birth (s-PTB) more accurately than standard ultrasound. Cervical cerclage, often performed to delay delivery, is sometimes difficult with standard 2-bladed speculums. The Bouquet Speculum^™^ combined with AI-assisted ultrasound offers the potential for improved outcomes and reduction of mortality and morbidity associated with preterm births, particularly in resource-limited settings. The Bouquet Speculum^™^ has demonstrated preliminary effectiveness in enhancing cervical visibility, patient comfort, and usability during cervical cerclage, a procedure employed to address preterm births associated with a short cervix or other cervical abnormalities. AI-assisted ultrasound enables early detection of s-PTB risk, allowing timely intervention with cervical cerclage to help prevent preterm births and associated infant and maternal complications. By integrating AI-assisted ultrasound with the Bouquet Speculum^™^, it is possible to overcome the limitations of traditional speculums and standard ultrasound and provide accurate, practical, effective, affordable, and safe solutions for reducing premature births. This method draws attention to a preventable cause of preterm birth and helps reduce mortality and morbidity while promoting better health for mothers and newborns, particularly in low-resource environments.

## Introduction

The Centers for Disease Control (CDC) and the World Health Organization (WHO) define preterm birth (PTB) as delivery of an infant prior to 37 weeks of gestation (1, 2). In 2020, 9.9% of infant deaths were attributed to PTBs (2). Additionally, complications of prematurity can lead to long-term morbidity (1). Globally, one in ten births is premature with nearly a million babies dying each year from complications of being born too soon (3). Currently, PTB’s are screened using history, physical exam, fetal fibronectin (fFN) levels, and/or cervical length (CL) measurements using ultrasound (4). Cervical cerclage, a purse-string stitch around the cervical os or opening, is often used to prevent preterm dilation. This procedure is sometimes difficult to perform even by the most skilled providers with 2-bladed speculums due to limited visibility of the cervix and other factors (5). Few AI programs have been implemented for obstetrics currently despite the benefit of AI’s application to prenatal and obstetric care. AI-assisted point of care ultrasound (AI-POCUS) can predict delivery timing and identify women at risk for PTB (6). In several clinical and simulated studies, the Bouquet vaginal speculum improved visibility of the cervix and facilitated gynecologic procedures such as cervical cerclage, although it should be noted that industry-sponsored device studies report systematically more favorable outcomes (7–11). This perspective article proposes that the Bouquet Speculum^™^, used alongside AI-POCUS, is a cost-effective, efficient, practical, and safe model to improve maternal and neonatal outcomes, especially in resource-constrained communities where the prevalence of PTBs is higher (2).

## Global economic burden of premature births

There were over 13 million babies born prematurely in 2020 (2), costing billions of dollars in acute care in neonatal intensive care units and creating financial and resource burdens on healthcare systems. The long-term individual costs of prematurity include negative impacts on earning potential, employment, and educational attainment. Additionally, the individual inherits lifelong health issues such as respiratory and cognitive diseases that are associated with being born preterm. This leads to lost workdays and a reduction in economic output. Resource constrained countries disproportionately shoulder this burden (2). AI-POCUS and the Bouquet Speculum^™^ enable point-of-care use, eliminating the need for referral to remote and expensive tertiary centers (8).

### A newly designed vaginal speculum

**Figure 1:**
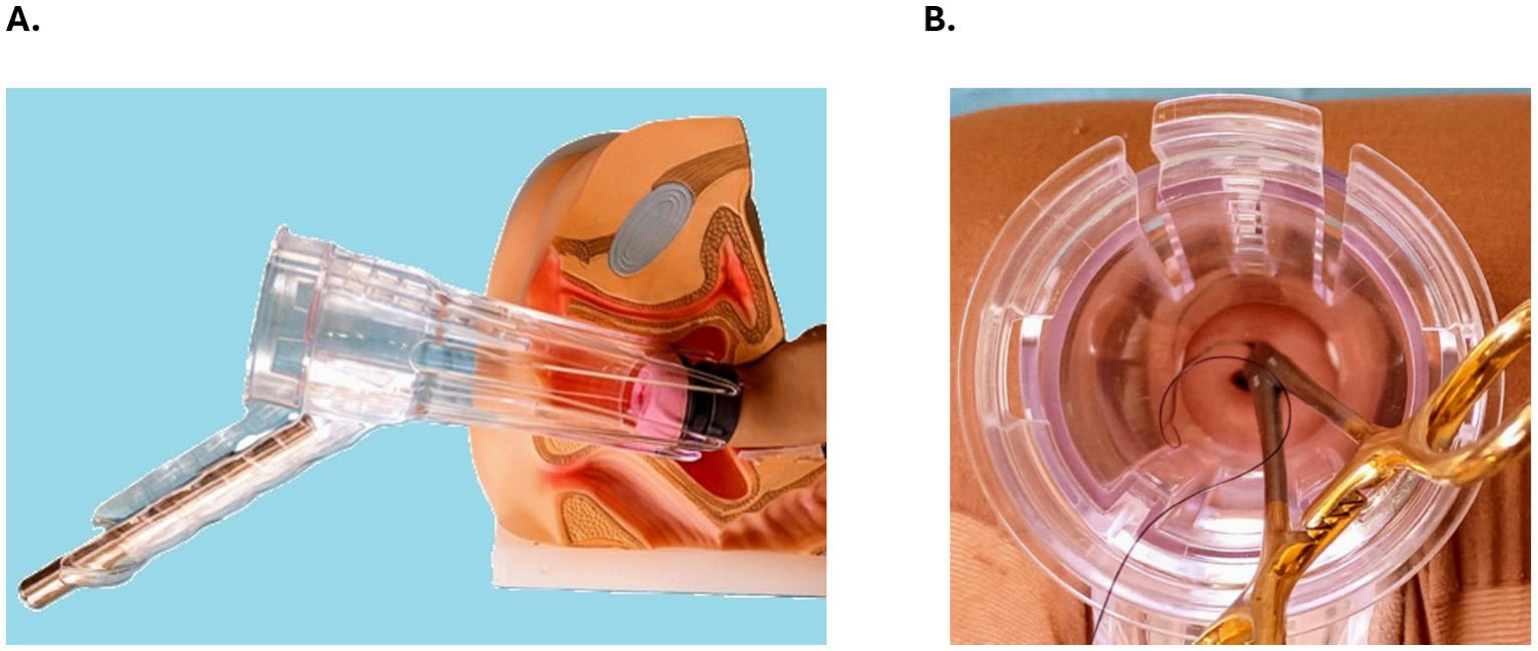
**(A)** The FDA-cleared, CE-marked Bouquet Speculum^™^ with a small insertive end, wide proximal opening for instrumentation, an oblique handle angle for use on any horizontal surface, a handle slot to accept a common penlight, and five petals that open in a radial fashion and cup the cervix for improved visualization and patient comfort. **(B)** Cervical cerclage simulation with suture and a long-handled needle driver through the Bouquet Speculum^™^. Photograph courtesy of the author, Jean M. Bouquet, DO.

Improved visualization of the cervix can help patients by reducing discomfort during examinations, enhancing the accuracy of cancer screening, and avoiding the need to perform the exam in an operating room under general anesthesia (12). According to a pilot survey study involving 100 participants, the Bouquet Speculum^™^ (Figure 1A) was associated with better cervical visualization, greater provider ease of use, and less patient discomfort when compared to standard speculums (10). Additionally, anatomical factors such as a retroverted uterus, lax lateral vaginal walls due to multiparity or obesity, and the presence of inflammation or infection can make cervical cerclage placement more challenging and increase the risk of introducing infection, causing bleeding, or prematurely rupturing the membranes (13). The potential benefits of the novel Bouquet Speculum^™^ may mitigate some of the limitations of the existing two-bladed speculum. For instance, better visibility and ease of use equate to more efficient and successful cervical cerclage outcomes (5).

**Figure 2:**
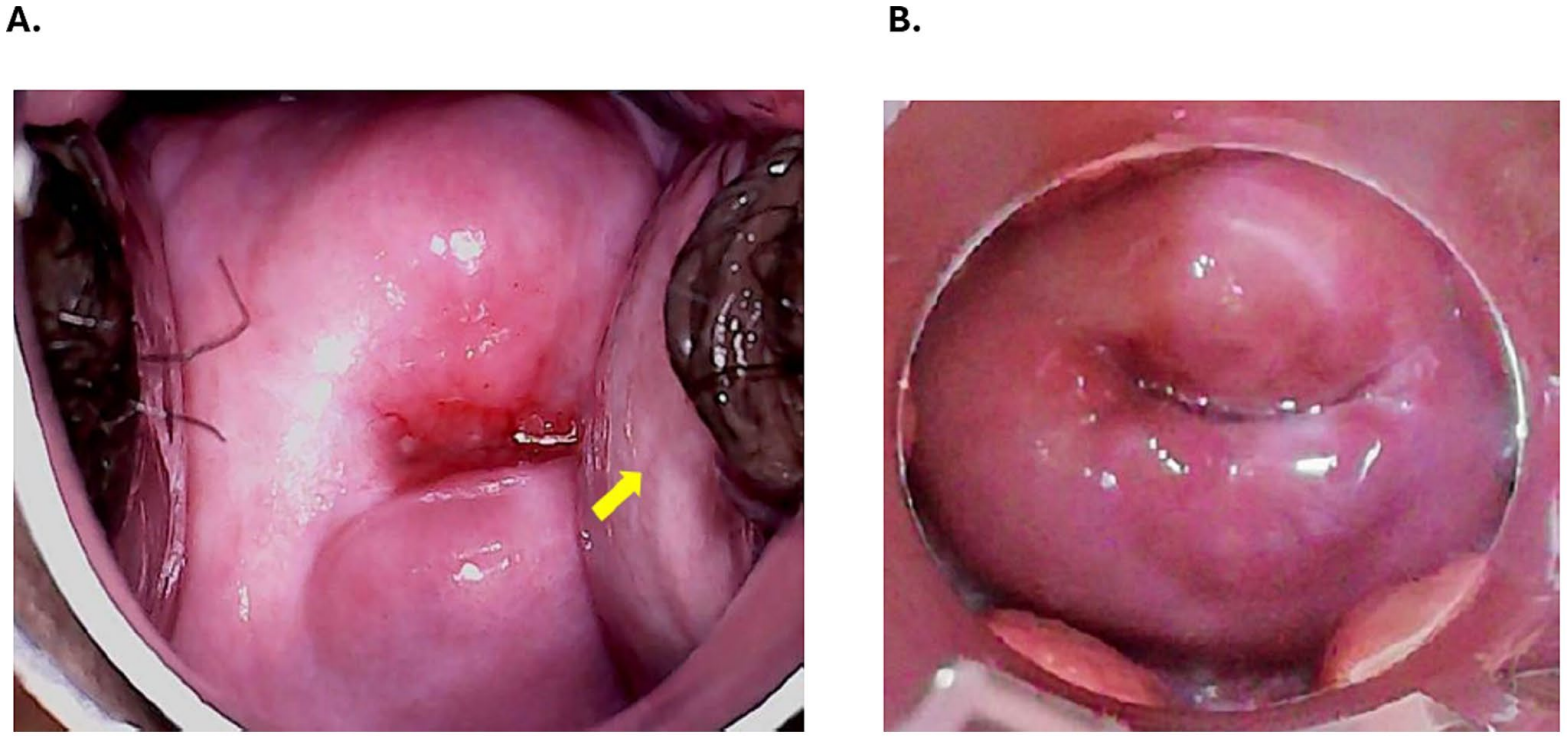
**(A)** Incomplete visualization of the cervix using a traditional two-bladed speculum with lateral vaginal wall collapse (yellow arrow) partially obscuring the complete cervical view. **(B)** Complete visualization of the cervix using the Bouquet Speculum^™^ without lateral vaginal wall collapse. Clinical photograph of the cervix. Photograph courtesy of Daniel Kimani, MD, Global Cancer Care and Research Institute.

Figure 1B demonstrates a simulated cervical cerclage procedure through the Bouquet Speculum^™^. The potential for improved ease of use and better visibility of the cervix with the Bouquet Speculum^™^ may also allow for a more efficient exam and treatment by less-experienced providers who may struggle with this procedure, such as those in low-resource settings (Figures 2A,B) (8, 10). The new 5-petaled vaginal speculum has a unique design that opens in a radial fashion and distributes the intravaginal forces equally (9). A pilot survey study reported less patient discomfort which may translate to less painful vaginal speculum exams and associated procedures (10).

### Screening

In 2023, machine learning-based prediction models for preterm birth screening showed poor reporting and methodological quality (14). Additionally, the ultrasound image dataset for machine learning shows biases that should be addressed (15). By diversifying data sources and populations, and improving models to better reflect population differences, the reliability of deep learning algorithms in fetal ultrasound analysis can be increased (16). Artificial intelligence has made significant progress over the last few years. A 2024 study found that deep learning models can extract features for assessing preterm birth more accurately than traditional visual methods (17). A major multicenter study showed that AI is more sensitive than cervical length measurement in detecting spontaneous preterm births across a range of characteristics, 19 different hospital sites, and various ultrasound machines. The AI model excels especially at earlier stages of pregnancy, allowing for timely preventive interventions. AI-assisted ultrasound has significantly enhanced PTB risk assessment, making it more accurate than traditional ultrasound methods (18). At the population level, universal transvaginal ultrasound screening of cervical length prior to 24 weeks’ gestation in singleton pregnancies without a previous spontaneous preterm birth is associated with a significant reduction in spontaneous preterm birth before 37 weeks compared with no screening (19). However, consensus is lacking regarding universal cervical length (CL) screening among asymptomatic women without a prior history of spontaneous preterm birth (sPTB). This gap supports the rationale of the present article to further develop and implement a universal artificial intelligence—assisted model for CL screening (20). AI-POCUS can detect subtle anatomical and physiological signs of premature aging or inflammation in reproductive structures, enhance cervical length measurement, provide better delivery predictions, and improve placental and amniotic fluid monitoring (21–23). Portable AI-enabled devices also expand access to areas with high PTB rates (21).

One scoping review noted significant improvements with the use of machine learning models in predicting obstetric outcomes, demonstrating the potential of AI to improve prenatal care and reduce maternal and child risks (14). They noted the ethical challenges regarding transparency and digital inequalities as most studies are not performed in underserved settings (14). However, therein lies opportunity to generate standardized datasets that can be shared between health professionals and administrators (15). This perspective also suggests that in combination with portable AI-enabled devices, the Bouquet Speculum^™^ can bridge the gap between higher resource technologies and areas with limited resources.

The authors reviewed the literature on the implications of AI-POCUS for predicting PTB. A study conducted in Zambia and North Carolina demonstrated that, even when an ultrasound probe was blindly swept across the gravid abdomen, AI-POCUS estimated gestational age with accuracy comparable to that of trained sonographers performing standard fetal biometry. Importantly, these findings highlight the potential of AI-POCUS to expand equitable access to high-quality obstetric imaging by enabling reliable assessments using blind sweeps acquired by untrained personnel with low-cost, portable devices in resource-limited settings such as Zambia (24, 25).

### Treatment

Cervical insufficiency, formerly referred to as cervical incompetence, represents a multifaceted syndrome frequently associated with preterm births (PTBs). Historically, management has involved empirical use of cervical cerclage. AI-assisted ultrasound improves diagnosis and management of PTB risk factors including genetics, anatomy, hormones, or inflammation from subclinical infections (14). Most deliveries before 34 weeks of gestation occur in individuals with no previous history of preterm birth. Midtrimester cervical length (CL) assessment using transvaginal ultrasound is one of the best clinical predictors of spontaneous preterm birth. Midtrimester cervical length of ≤25 mm is recommended to diagnose short cervix in singleton pregnancies without prior spontaneous preterm birth (26). According to one study, choosing cervical cerclage for patients with a history of PTB was costlier and offered slightly less effectiveness compared to the transvaginal ultrasound cervical length assessment strategy, resulting in a lower net monetary benefit (27). After hand-held AI-POCUS has been performed and it is determined that there is a short cervix or other cervical remodeling changes that warrant treatment, a relatively simple cervical cerclage can be performed. Cervical cerclage has been used for 60 years to manage women at risk of preterm birth, but many questions remain. While research supports its use in some cases, especially singleton pregnancies, questions about adjunct therapies and effectiveness for high-risk subgroups remain unresolved. Large RCTs are needed to identify which patients benefit most and the best methods for the procedure (28, 29). Adjunctive therapy combining cervical cerclage with vaginal progesterone may be associated with a greater reduction in preterm birth compared with either intervention alone. However, these findings should be interpreted cautiously, and further well-designed, adequately powered randomized controlled trials are required to confirm the efficacy and clinical utility of this combined therapeutic approach (30).

The Bouquet Speculum^™^ may enhance AI-POCUS and cervical cerclage outcomes in neonatal and obstetric care by improving cervical visualization, ease of use, and patient comfort (8–10). The radially expanding design of the Bouquet Speculum allows circumferential engagement of the cervix, facilitating controlled anterior traction and centering within the visual field (9). This may improve access in cases of high, posterior, or anatomically challenging cervices (9). Radial dilation provides uniform circumferential exposure of the cervix without preferential compression in the anterior–posterior plane. This can reduce reliance on additional vaginal wall retractors to visualize posterior and lateral cervical tissue (8–10). By gently clasping the cervix, the device may reduce cervical mobility during needle passage. Improved stabilization can support more symmetric tissue bites and higher stitch placement, particularly in exam-indicated or rescue cerclage. Integrated visualization and stabilization may decrease the need for weighted posterior speculums or multiple retractors. This has the potential to simplify procedural setup and reduce operative or in-office procedural complexity. The adaptable radial expansion accommodates variability in vaginal anatomy, including increased vaginal wall pressure or cervical position (9). This may enhance exposure in patients where standard bivalve speculums provide limited visualization. Radial expansion distributes pressure circumferentially rather than concentrating force across opposing blades (9). This may reduce tissue distortion around the cervix during exposure. The device’s ability to provide stable, circumferential exposure and cervical control aligns with key technical objectives emphasized in cerclage literature. The design directly addresses recognized limitations of traditional bivalve exposure strategies (8–10).

## Discussion

All published clinical data regarding the Bouquet Speculum^™^ to date have been generated or co-authored by its inventors or investigators affiliated with the originating institution; independent studies conducted by unaffiliated research groups have not yet been published. Although this necessarily limits the current evidentiary base supporting broader integration of the Bouquet Speculum^™^, it would be neither scientifically sound nor ethically appropriate to withhold potentially practice-informing innovations that may improve outcomes for mothers and infants at risk of preterm birth.

This perspective article further acknowledges that the proposed approach integrates a novel, preliminarily validated vaginal speculum with an emerging AI-assisted ultrasound model, together offering the potential to reduce both neonatal and maternal morbidity and mortality associated with preterm birth. Notably, the most substantial challenge lies in the successful implementation and sustainable deployment of this combined model, particularly within resource-constrained settings. Critical considerations include long-term sustainability, in-country regulatory requirements, potential biases inherent to machine-learning algorithms, and the generalizability of the model across diverse clinical settings and populations (31–33).

By integrating AI-assisted ultrasound with the Bouquet Speculum^™^, it is possible to overcome the limitations of traditional specula and standard ultrasound and provide point of care, accurate, effective, affordable, and safe solutions for reducing premature births. This method draws attention to a preventable cause of preterm birth and helps reduce mortality and morbidity while promoting better health for mothers and newborns, particularly in low-resource environments. Future efforts will be directed at researching and implementing this combination of technologies for real-world impact and the positive outcome of reducing the number of infants that are born too soon.

## Data Availability

The original contributions presented in the study are included in the article/supplementary material, further inquiries can be directed to the corresponding authors.
